# Integrated polyphasic characterization and mycotoxin production of fungal isolates in sugarcane (*Saccharum officinarum*) stems from Thailand

**DOI:** 10.3389/fnut.2026.1828952

**Published:** 2026-06-10

**Authors:** Chonlada Doungsrikaew, Amnart Poapolathep, Donnaya Thanakitpipattana, Nattawut Boonyuen, Phattarawadee Wattanasuntorn, Onuma Piasai, Johanna Fink-Gremmels, Xianfeng Ren, Antonio F. Logrieco, Saranya Poapolathep

**Affiliations:** 1 Department of Pharmacology, Faculty of Veterinary Medicine, Kasetsart University, Bangkok, Thailand; 2 National Center for Genetic Engineering and Biotechnology (BIOTEC), National Science and Technology Development Agency (NSTDA), Pathum Thani, Thailand; 3 Department of Plant Pathology, Faculty of Agriculture, Kasetsart University, Bangkok, Thailand; 4 Institute for Risk Assessment Sciences, Faculty of Veterinary Medicine, Utrecht University, Utrecht, Netherlands; 5 Institute of Agricultural Quality Standards and Testing Technology of Shandong Academy of Agricultural Sciences, Jinan, China; 6 Xianghu Laboratory, Zhejiang Provincial Laboratory of Agriculture, Hangzhou, China; 7 Research National Council, Institute of Sciences of Food Production, Bari, Italy

**Keywords:** fungal diversity, LC–MS/MS, mycotoxin-producing fungi, mycotoxins, sugarcane

## Abstract

Sugarcane (*Saccharum officinarum*) is a major agro-industrial crop in Thailand, whose sucrose-rich stems provide a favorable ecological niche for colonization by diverse fungal taxa, including species capable of producing harmful mycotoxins. Despite its importance, integrated assessments of fungal diversity and toxigenic potential in Thai sugarcane tissues remain limited. In this study, we applied an integrated polyphasic approach combining morphological characterization, multilocus phylogenetic analyses, and targeted liquid chromatography–electrospray ionization–tandem mass spectrometry (LC–ESI–MS/MS) to characterize fungi isolated from sugarcane stems collected in Kanchanaburi Province, Thailand. A total of 64 isolates were assigned to nine genera, with *Curvularia* (57.81%), *Fusarium* (15.62%), and *Nigrospora* (9.38%) predominating. Multilocus sequence analyses based on the internal transcribed spacer region (ITS), translation elongation factor 1-alpha (TEF1-*α*), RNA polymerase II second largest subunit (RPB2), *β*-tubulin (TUB2), glyceraldehyde-3-phosphate dehydrogenase (GAPDH), and calmodulin (CAM) resolved representative isolates into *Curvularia plantarum*, *C.* cf. *guangxiensis*, *C.* cf. *clavata*, *Fusarium sacchari*, *F. pernambucanum*, *F. chlamydosporum*, and *Nigrospora zimmermanii*. LC–ESI–MS/MS analysis revealed that all *F. sacchari* isolates were capable of producing beauvericin, whereas *Paecilomyces lecythidis* synthesized citrinin. Notably, this study provides, to the best of our knowledge, the first evidence of citrinin biosynthesis by *P. lecythidis* and represents the first integrated polyphasic evaluation of fungal diversity and mycotoxin-producing potential in Thai sugarcane stems. These findings highlight the presence of field-associated toxigenic fungi and suggest potential risks to food and feed safety, underscoring the need for surveillance strategies across the sugarcane production and processing chain.

## 1 Introduction

Sugarcane (*Saccharum officinarum* L.) is a member of the family Poaceae, tribe Andropogoneae, which also includes other economically important crops such as maize and sorghum (1). As a C4 plant, sugarcane possesses an efficient carbon fixation mechanism that supports high productivity under elevated temperatures (1–3). This physiological advantage has contributed to its extensive cultivation across tropical and subtropical regions in more than 100 countries worldwide (2, 4). A defining feature of sugarcane is its remarkable ability to accumulate high concentrations of sucrose in the stem (5), making it the primary raw material for approximately 80% of global sugar production (6). According to FAOSTAT, Thailand ranked as the fourth-largest sugarcane producer globally (7), with major sugarcane-producing provinces such as Kanchanaburi, Nakhon Sawan, Khon Kaen, Kamphaeng Phet, and Udon Thani (8, 9). Beyond sugar production, the industry generates valuable by-products such as molasses, bagasse, and filter cake, which support diverse industries, including ethanol production, animal feed, bioelectricity, particleboard manufacturing, composting, fertilizer production, and rum distillation (10–12).

However, the high sugar content and fibrous internal tissues of sugarcane stems create a nutrient-rich ecological niche that facilitates colonization by diverse saprobic, endophytic, and pathogenic fungi. These fungi not only contribute to deterioration of stalk quality but may also synthesize biologically active secondary metabolites, including mycotoxins that have implications for food safety and industrial processing (13, 14). Numerous fungal taxa have been reported as causal agents of major sugarcane diseases worldwide, including *Colletotrichum, Fusarium, Phoma, Cochliobolus, Nigrospora, Bipolaris, Curvularia,* and *Macrophomina*, each associated with distinct symptomatologies (15, 16). These diseases can lead to lower sucrose content, reduced yield, and poor overall quality, resulting in significant economic losses (17). Furthermore, storage fungi such as *Aspergillus* and *Penicillium* are capable of producing mycotoxins (18). Favorable environmental conditions in the field—particularly high temperature and humidity—can promote mycotoxin production during plant growth, whereas inadequate storage conditions further increase post-harvest contamination risks (19). Mycotoxin contamination has been widely reported in various agricultural commodities, with potential adverse health effects ranging from toxic effects on multiple organs to carcinogenic effects (20). This challenge is compounded by the co-occurrence of multiple mycotoxins, which can lead to additive, synergistic effects, and in some cases antagonistic effects (21), and their thermal stability during food and feed processing processes. Thus, mycotoxin contamination represents a common challenge for food and feed safety (22). Despite extensive studies on mycotoxin prevalence in other crops, research on mycotoxin contamination associated with sugarcane production is limited. Available studies have documented various contamination risks, ranging from the isolation of mycotoxin-producing fungi from sugarcane such as *Aspergillus* spp. and *Fusarium* spp. (13, 23) to confirmed contamination in sugarcane products like juice and molasses (24). While these reports highlight risks to food and feed safety, in Thailand, research on mycotoxin issues in sugarcane is notably scarce, with available studies focusing only on general fungal incidence in bagasse and filter cake (25). Therefore, this study aimed to: (1) investigate fungi isolated from sugarcane samples collected in Kanchanaburi Province, Thailand; (2) determine their evolutionary relationships using molecular phylogenetic analyses; and (3) analyze their mycotoxin-producing potential.

## 2 Materials and methods

### 2.1 Sample collection and fungal isolation

Ten sugarcane stems of cultivar Khon Kaen 3 at the mature stage (approximately 12 months old) were randomly collected from a single large-scale commercial sugarcane field in Kanchanaburi Province, Thailand, in December 2023. A simple random sampling approach was used, with stems collected from various points across the field. After sampling, the stems were stored at 4 °C until further analysis. Fungal isolation was performed using a tissue transplantation technique, with minor modifications to the method described by Agrios (26). The outer rind of each stem was aseptically removed using a sterile knife, and the inner parenchymatous tissues were cut into small pieces. The tissue pieces were surface sterilized with 0.5% (v/v) sodium hypochlorite solution, prepared from 10% (v/v) commercial bleach (Clorox), for 5 min, rinsed with sterile distilled water and dried on sterile filter paper. Sterilized tissue pieces were transferred onto potato dextrose agar (PDA, Difco, Detroit, MI, USA) plates, maintaining equal spacing (four pieces per plate) and incubated at 25 °C for 7 days to allow fungal growth.

### 2.2 Chemicals and reagents

OmniPCR Supermix (OnePCR™ Ultra) and OneMARK 100 DNA ladder were purchased from Bio-Helix (New Taipei, Taiwan). PCR primers were synthesized by Macrogen (Seoul, South Korea). Cetyltrimethylammonium bromide (CTAB), chloroform, isoamyl alcohol, isopropanol, 70% ethanol, TE buffer, and agarose were of analytical grade. Milli-Q water was prepared using a purification system from Millipore (Bedford, MA, USA). Acetonitrile, methanol, and formic acid (HPLC grade) were purchased from RCI Labscan (Bangkok, Thailand). Ammonium formate was purchased from Sigma-Aldrich (Taufkirchen, Germany). Magnesium sulfate (MgSO₄) was purchased from PanReac Química (Barcelona, Spain). Sodium chloride (NaCl) was purchased from KemAus (New South Wales, Australia). Octadecyl-bonded silica (C18) was purchased from Macherey-Nagel (Düren, Germany), and primary–secondary amine (PSA) was purchased from Biocomma Biotech (Shenzhen, China).

### 2.3 Preliminary morphological identification

Fungal colonies emerging from the stem tissues were subcultured onto fresh PDA plates by transferring small agar blocks and incubating them in a controlled chamber at 25 °C for 7 days to obtain pure cultures. Colony characteristics, including the color of the obverse and reverse sides and growth rate were recorded. Spore morphology, such as spore shape and size was examined under a light microscope equipped with an Axiocam 208 color camera (Carl Zeiss, Jena, Germany) at 400 × magnification (27–29). For isolates identified as belonging to the genus *Fusarium*, isolates were cultured on carnation leaf agar (CLA), which was prepared using sterile carnation leaf fragments placed on agar, following Fisher et al. (30), and incubated for 14 days to observe macroconidia, microconidia, and chlamydospores (31). Following morphological observations, isolates within each genus were categorized into distinct morphotypes. Representative isolates from each morphotype were then selected for multilocus phylogenetic analysis to achieve species-level identification.

### 2.4 DNA extraction, PCR amplification and sequencing

Fungal strains were cultured on PDA and incubated at 25 °C for 7 days. Total genomic DNA was extracted from fungal mycelia using the cetyltrimethylammonium bromide (CTAB) method (32, 33). Mycelia were scraped from the culture plates, transferred to microtubes, and frozen prior to extraction. Approximately 600 μL of CTAB buffer was added to each sample and homogenized using a sterile plastic pestle. The homogenate was incubated at 65 °C for 1 h using a Digital Dry Bath (Thermo Fisher Scientific, Waltham, MA, USA), followed by the addition of 600 μL of chloroform: isoamyl alcohol (24:1, v/v). After gentle mixing, the samples were centrifuged at 12,000 rpm for 10 min using a 5415 R microcentrifuge (Eppendorf, Hamburg, Germany), and the resulting supernatant was transferred to a new microtube. DNA precipitation was performed by adding 300 μL of chilled isopropanol and centrifuging at 12,000 rpm for 5 min. The DNA pellet was washed with 1 mL of 70% (v/v) ethanol, air-dried, and dissolved in 50 μL of Tris-EDTA (TE) buffer (32). DNA concentration and purity were determined using a NanoDrop OneC spectrophotometer (Thermo Fisher Scientific, Waltham, MA, USA). The extracted DNA served as a template for PCR amplification using a T100 Thermal Cycler (Bio-Rad, Hercules, CA, USA). Target gene regions, including internal transcribed spacer (ITS), RNA polymerase II second largest subunit (RPB2), *β*-tubulin (TUB), calmodulin (CAM), translation elongation factor 1-alpha (TEF1-*α*), and glyceraldehyde-3-phosphate dehydrogenase (GAPDH), were amplified using the gene-specific primer pairs (ITS4/ITS5, 5F2/7cR, Bt2a/Bt2b or T1/Bt2b, CL1/CL2a, cmd5/cmd6, EF1/EF2, 983F/2218R, 728F/EF2, and GPD1/GPD2), depending on the fungal genus (15, 23, 34–40). PCR products were verified by electrophoresis on a 1.5% (w/v) agarose gel at 100 V for 25 min using an Mupid-exU electrophoresis system (Advance, Tokyo, Japan) and visualized under ultraviolet light using an SLB-01 W UltraSlim LED transilluminator (MaestroGen, Hsinchu, Taiwan) to confirm expected band sizes. Amplified products were then submitted for Sanger sequencing to commercial providers, including Macrogen (Seoul, South Korea) and Apical (Selangor, Malaysia).

### 2.5 Sequence editing, alignment, and phylogenetic analysis

Sequences obtained from each gene locus were assembled and edited using BioEdit v7.7.1 (2021) (41). Each locus was subjected to a BLAST search against the NCBI database to identify and retrieve closely related representative sequences for phylogenetic comparison (42). Accession numbers of all reference strains used in this study are provided in Supplementary Table S1. Reference sequences were selected based on previously published taxonomic and phylogenetic studies relevant to each fungal genus (15, 35, 43–50). Multiple sequence alignments for each locus were performed using the MUSCLE algorithm and manually adjusted where necessary (51). Following individual alignments, loci were concatenated to generate a combined dataset for multilocus phylogenetic analysis. Maximum likelihood (ML) phylogenetic trees were constructed using RAxML-HPC2 (v8.2.12) on XSEDE via the CIPRES Science Gateway (52), employing the GTR + GAMMA substitution model. ML tree searches were performed with 1,000 rapid bootstrap replicates using the RAxML rapid bootstrapping algorithm. An appropriate outgroup taxon was designated to root each tree. Aligned datasets were then exported in NEXUS format using PAUP v4.0a for Bayesian inference (BI) analysis (53). BI analyses were performed in MrBayes v3.2.7, running 5,000,000 Markov chain Monte Carlo generations, with the first 5,000 generations discarded as burn-in. Posterior probability values were used to assess branch support (54). Resulting consensus trees were visualized using FigTree v1.4.4. Phylogenetic tree construction and species identification followed the methodological framework outlined by Raja et al. (55). For isolates occurring at low frequency, species identification was confirmed by BLAST analysis using sequences from at least three gene loci (56).

### 2.6 Fungal culture extraction for mycotoxin analysis

The extraction process was performed following the method of Seo et al. (57) with minor modifications. Briefly, the incubation period was adjusted for each fungal genus, with cultures grown for different durations based on published literature on mycotoxin production. Five grams of fungal culture were acidified with 1 mL of 10% formic acid and allowed to stand for 30 min. Then, 9 mL of acetonitrile was added, and the mixture was shaken for 30 min using an orbital shaker (Arthur H. Thomas, Philadelphia, PA, USA). Subsequently, 4 g of magnesium sulfate and 1 g of sodium chloride were added, followed by vortex mixing for 1 min and centrifugation at 4,000 rpm for 10 min using an Allegra X-30R centrifuge (Beckman Coulter, Brea, CA, USA). After centrifugation, 1 mL of the supernatant was subjected to clean-up using a d-SPE (C18, 25 mg; PSA, 25 mg). The mixture was vortexed and centrifuged again at 10,000 rpm for 5 min. Finally, 400 μL of the obtained supernatant was diluted with 500 μL of Milli-Q water and 100 μL of acetonitrile, filtered through a 0.22 μm nylon syringe filter (HAMAG, Ningbo, China), and injected into the LC–MS/MS system for analysis.

### 2.7 LC-(ESI)-MS/MS conditions and method validation

The LC–MS/MS analysis was conducted following procedures that were adapted from earlier publications (38, 58, 59). The analytical column was an Eclipse Plus C18 1.8 μm (4.6 × 50 mm), maintained at 40 °C during analysis. The mobile phase consisted of 5 mM ammonium formate with 0.5% formic acid in Milli Q (Mobile phase A) and methanol (Mobile phase B) using a gradient elution program. The gradient elution program was as follows: 0–1 min, 90% A and 10% B; 1–5 min, linear decrease of A from 90 to 5% and increase of B from 10 to 95%; 5–10 min, held at 5% A and 95% B; 10–10.2 min, returned to 90% A and 10% B; 10.2–15 min, equilibrated at 90% A and 10% B. The flow rate of mobile phase was 0.4 mL/min. The injection volume was 10 μL. Mass spectrometric detection was performed using a triple quadrupole mass analyzer (6460 triple, Agilent Technologies, Waldbronn, Germany) with electrospray ionization (ESI) operated in both positive and negative ion modes under multiple reaction monitoring (MRM) mode to detect target analytes. The specific transitions for the target analytes are listed in Supplementary Table S2.

### 2.8 Method validation

The analytical method was validated for targeted mycotoxins, including beauvericin (BEA), fumonisin B1 (FB1), fumonisin B2 (FB2), enniatin A (ENN A), enniatin A1 (ENN A1), enniatin B (ENN B), enniatin B1 (ENN B1), T-2 toxin, zearalenone (ZEA), nivalenol (NIV), deoxynivalenol (DON), sterigmatocystin (STER), ochratoxin A (OTA), patulin (PAT), citrinin (CIT), and alternariol (ALT) in accordance with Commission Decision 2002/657/EC (60). Identification of each analyte was confirmed by monitoring at least two specific compound MRM transitions, and the retention time was verified against the corresponding certified standard. The limits of detection (LOD) were determined at a signal-to-noise ratio of 3:1, while the limits of quantification (LOQ) were set at 10:1. The LOD values for all target analytes ranged from 0.02 to 25.6 μg/kg and the LOQ values ranged from 0.08 to 85.3 μg/kg in the culture media.

## 3 Results

### 3.1 Preliminary morphological identification, diversity and occurrence

**Figure 1:**
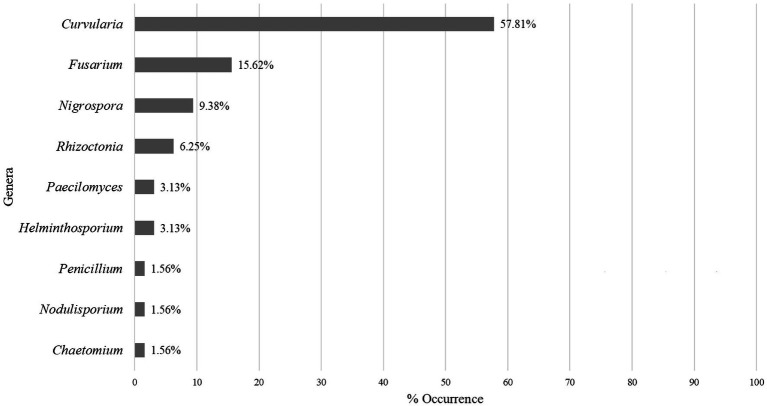
Percentage occurrence of fungal genera isolated from sugarcane stem tissues collected in Kanchanaburi Province, Thailand.

**Figure 2:**
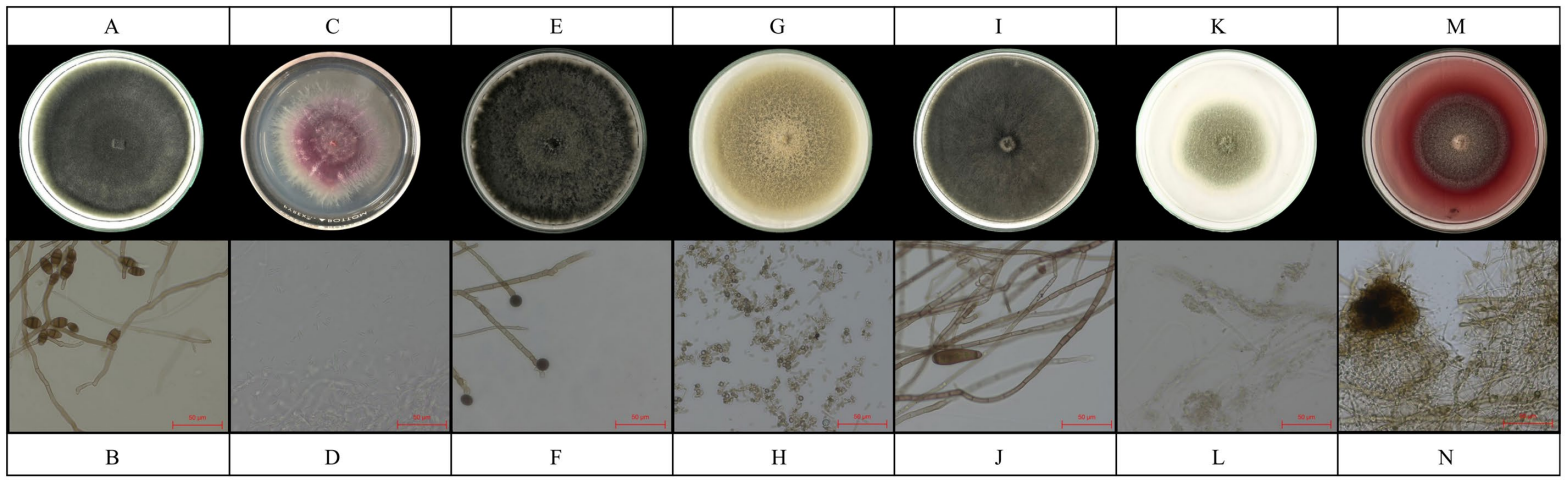
Colony morphology on PDA after 7 days and microscopic features showing spore morphology. **(A,B)** *Curvularia plantarum* SC53; **(C,D)** *Fusarium sacchari* SC11 (**D**: conidial morphology observed from CLA at 14 days); **(E,F)** *Nigrospora zimmermanii* SC23; **(G,H)** *Paecilomyces lecythidis* SC32; **(I,J)** *Exserohilum rostratum* SC13; **(K,L)** *Talaromyces funiculosus* SC39; **(M,N)** *Arcopilus cupreus* SC22. Scale bars: 50 μm.

Based on their morphological characteristics, the fungal isolates were classified into nine genera: *Curvularia*, *Fusarium*, *Nigrospora*, *Rhizoctonia*, *Paecilomyces*, *Helminthosporium*, *Nodulisporium*, *Penicillium*, and *Chaetomium*. Among these, the dominant genera were *Curvularia*, *Fusarium*, and *Nigrospora*. *Curvularia* represented the highest proportion of isolates (37/64, 57.81%), followed by *Fusarium* (10/64, 15.62%), and *Nigrospora* (6/64, 9.38%), as shown in Figure 1. Further classification within each dominant genus based on consistent colony and microscopic features showed three distinct major morphotypes for *Curvularia*, six for *Fusarium*, and two for *Nigrospora*. Representative colony morphology characteristics and microscopic features of the dominant genera, along with other noteworthy isolates, are presented in Figure 2. These results are consistent with previous studies reporting that these genera frequently occur in sugarcane, although most earlier reports noted isolates predominantly from leaves and roots rather than stems (15).

### 3.2 Multilocus phylogenetic analyses

**Figure 3:**
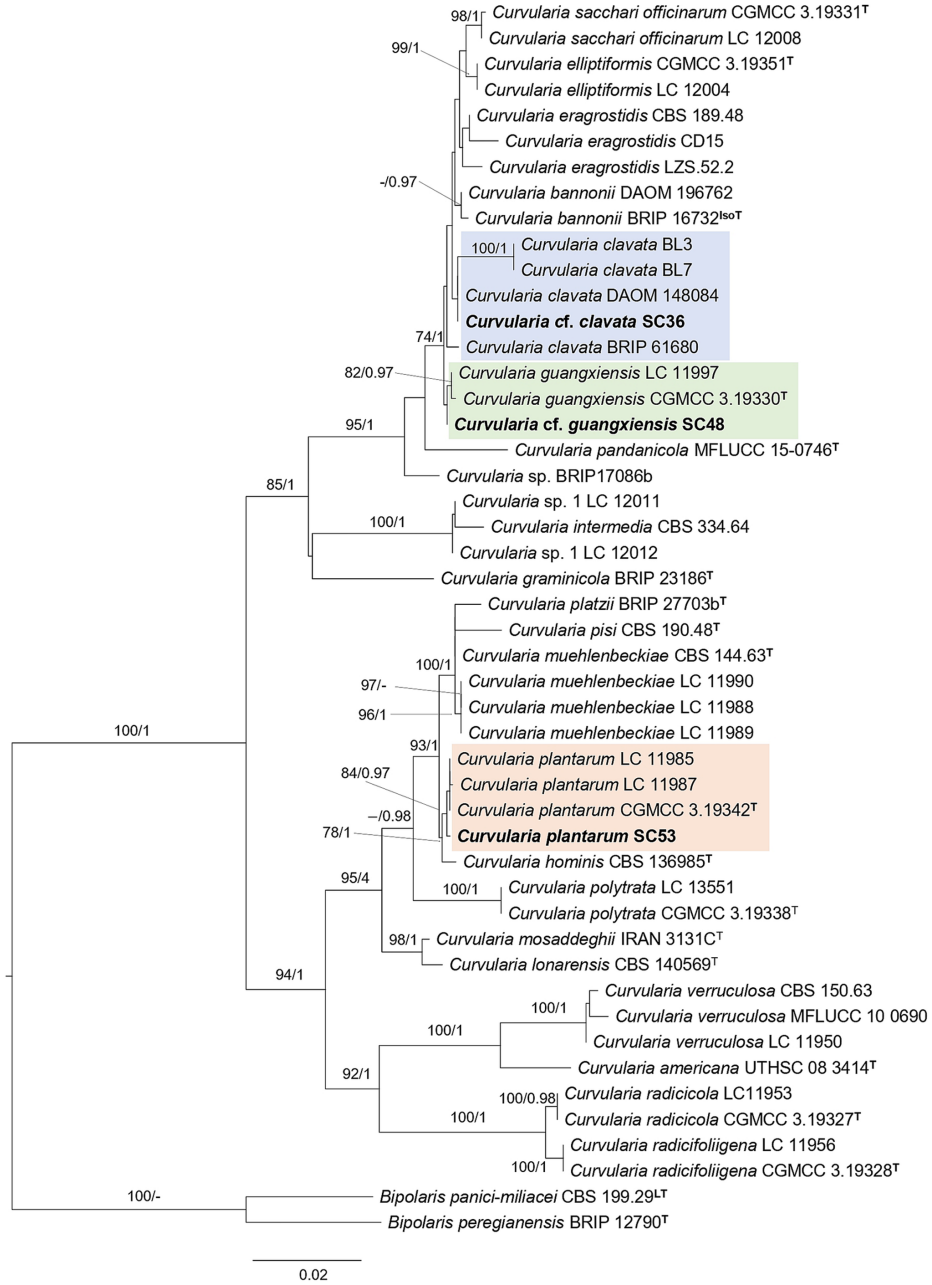
Maximum likelihood phylogenetic tree (1,000 bootstrap replicates) method based on combined ITS, TEF1-*α*, and GAPDH gene sequences of *Curvularia* isolates from sugarcane collected in Kanchanaburi Province, Thailand.

**Figure 4:**
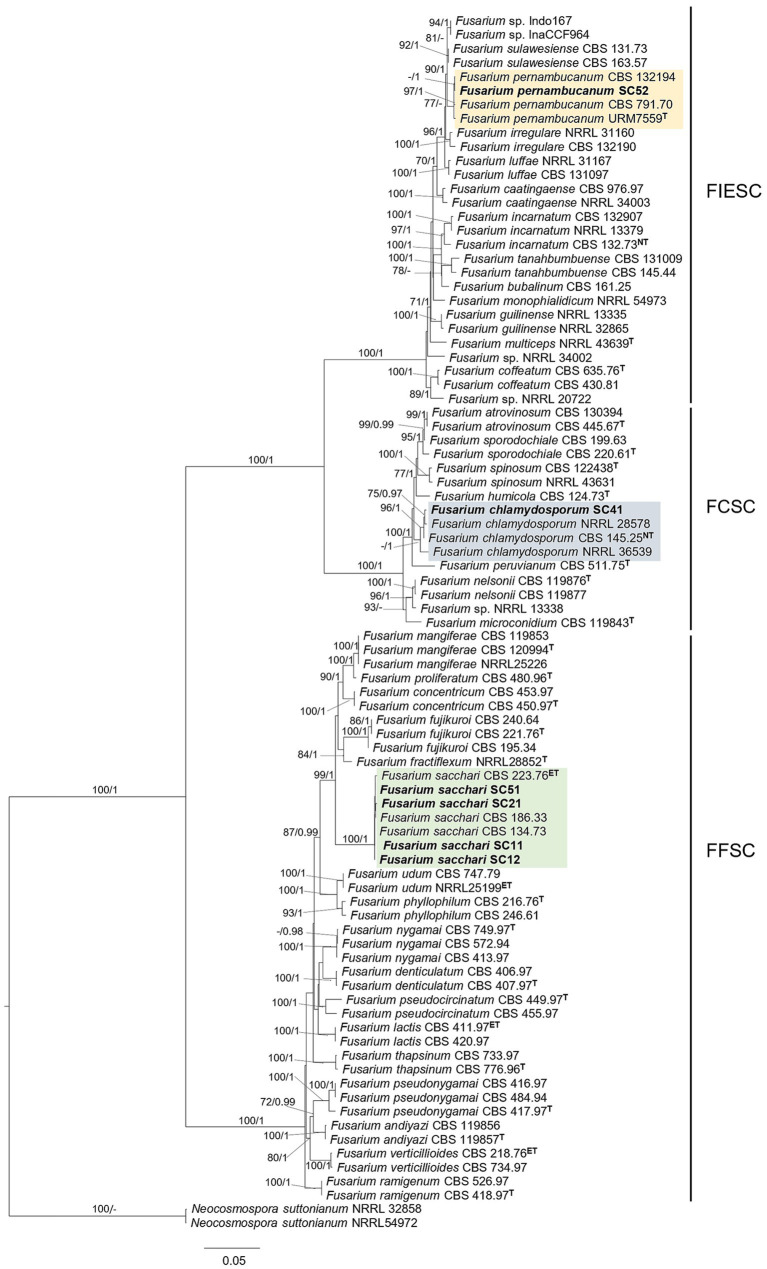
Maximum likelihood phylogenetic tree (1,000 bootstrap replicates) based on combined TEF1-α, RPB2, and CAM gene sequences of *Fusarium* isolates from sugarcane collected in Kanchanaburi, Thailand.

**Figure 5:**
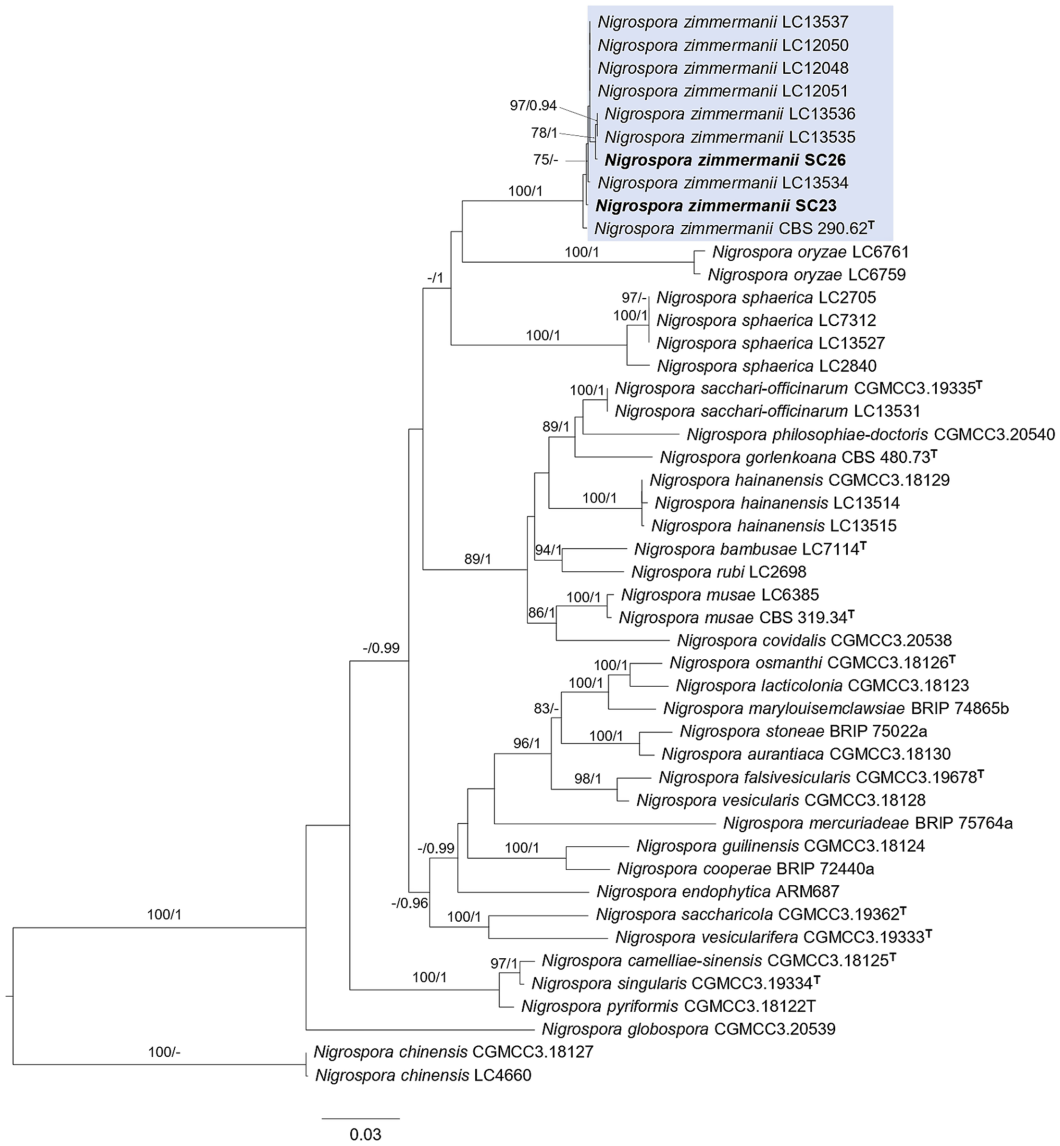
Maximum likelihood phylogenetic tree (1,000 bootstrap replicates) based on combined ITS, TEF1-α, and TUB gene sequences of *Nigrospora* isolates from sugarcane collected in Kanchanaburi, Thailand.

Molecular identification was carried out using multilocus sequence analyses. Phylogenetic analyses were performed for the dominant genera (*Curvularia*, *Fusarium*, and *Nigrospora*) by selecting representative isolates of each morphotype group to confirm species-level identifications and infer evolutionary relationships. For *Curvularia*, phylogenetic reconstruction based on a concatenated ITS, TEF1-*α*, and GAPDH dataset (2,172 aligned positions) resolved the three isolates into distinct species-level clades. The isolates were identified as *Curvularia plantarum*, *C.* cf. *guangxiensis*, and *C.* cf. *clavata*, as shown in Figure 3. Six *Fusarium* isolates were analyzed using a combined dataset of TEF1-α, RPB2, and CAM gene sequences (2,404 aligned positions). The resulting phylogenetic tree (Figure 4) placed four isolates within *Fusarium sacchari* (*F. fujikuroi* species complex, FFSC), one isolate within *F. pernambucanum* (*F. incarnatum*–*equiseti* species complex, FIESC), and one isolate within *F. chlamydosporum* (*F. chlamydosporum* species complex, FCSC). The two *Nigrospora* isolates were examined using a concatenated ITS, TUB, and TEF1-*α* gene dataset (1,558 aligned positions). Both isolates clustered within the *Nigrospora zimmermanii* clade, confirming their species identity (Figure 5). Isolates recovered at lower frequencies were identified using BLAST searches of individual gene sequences against the NCBI database. Species identifications were based on the closest matches with similarity values exceeding 97% for all loci analyzed. These BLAST-based identifications are summarized in Table 1.

**Table 1:** BLAST-based identification of fungal isolates from sugarcane using selected gene regions (ITS, TUB, CAM, TEF1-α, and RPB2), compared with the closest reference sequences in the NCBI.

Morphological identification	Gene	Accession no. (this study)	Closest match (accession no.)	Identity (%)
*Chaetomium* sp. SC22	ITS	PX494407	*Arcopilus cupreus* (MH861590)	99.83%
	RPB2	PX829176	*Arcopilus cupreus* (MZ342999)	99.10%
	TUB	PX829181	*Arcopilus cupreus* (KX976926)	98.84%
*Helminthosporium* sp. SC13	ITS	PX494404	*Exserohilum rostratum* (OR473124)	100%
	TEF1-α	PX829165	*Exserohilum rostratum* (HE664080)	99.79%
	RPB2	PX829174	*Exserohilum rostratum* (LT882524)	100%
*Paecilomyces* sp. SC32	ITS	PX494406	*Paecilomyces lecythidis* (PP191149)	98.71%
	TUB	PX829180	*Paecilomyces lecythidis* (PP197735)	98.37%
	CAM	PX829153	*Paecilomyces lecythidis* (PP197770)	99.69%
*Penicillium* sp. SC39	ITS	PX494403	*Talaromyces funiculosus* (MZ014550)	100%
	RPB2	PX829173	*Talaromyces funiculosus* (OR242035)	100%
	TUB	PX829179	*Talaromyces funiculosus* (MZ027969)	99.56%
	CAM	PX829152	*Talaromyces funiculosus* (OR242011)	99.79%

### 3.3 Mycotoxin analysis by representative isolates

**Figure 6:**
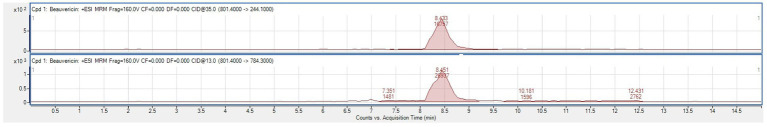
Chromatograms obtained by LC/MS–MS analysis of beauvericin produced by *Fusarium sacchari*.

**Figure 7:**
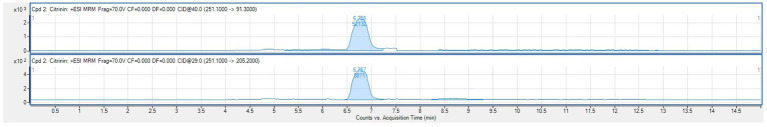
Chromatograms obtained by LC/MS–MS analysis of citrinin produced by *Paecilomyces lecythidis*.

Representative isolates were selected to evaluate their potential to produce mycotoxins based on published literature using LC–MS/MS. The performance characteristics of the analytical method, including LOD and LOQ, are detailed in Supplementary Table S3. Our results showed that all isolates of *Fusarium sacchari* (SC11, SC12, SC21, and SC51) grown on PDA at 25 °C in the dark for 14 and 21 days produced BEA (Figure 6). In addition, one isolate of *Paecilomyces lecythidis* (SC32) grown on PDA at 25 °C for 21 days exhibited the ability to produce CIT (Figure 7). The identity of these mycotoxins was rigorously confirmed by comparing their MS/MS transitions and retention times with certified standards. For the purpose of assessing the toxigenic potential of the isolates, these results are reported qualitatively. No other targeted mycotoxins were detected under the experimental conditions.

## 4 Discussion

The present study provides the first integrated polyphasic characterization of fungal isolates associated with sugarcane stems in Thailand and evaluates their capacity to produce selected mycotoxins. The combination of morphology, multilocus phylogeny, and LC–ESI–MS/MS toxin profiling enabled robust species delimitation and detection of toxigenic potential. This present study reveals predominant fungal isolates belonging to *Curvularia*, followed by *Fusarium*, and *Nigrospora*. Although comprehensive studies focusing on fungi inhabiting the parenchyma of sugarcane stem from other sugarcane-producing regions are limited, these fungal genera have been repeatedly reported to be associated with sugarcane across different tissues and ecological contexts. *Fusarium* is frequently isolated from sugarcane stems and leaves and is recognized as a destructive pathogen causing diseases such as wilt and pokkah-bong in several countries, including India, Brazil, China and Thailand (61–64). In contrast, *Curvularia* and *Nigrospora* have been widely documented in tropical and subtropical sugarcane-growing regions worldwide, primarily found in leaves and roots, where they are associated with endophytic colonization and foliar diseases (15, 65, 66). The finding of these genera across different tissues and geographic regions suggests that they are broadly associated with sugarcane rather than being restricted to specific tissue or local environments. The predominance of *Curvularia* in the parenchyma observed in the present study represents a notable shift from the commonly emphasized dominance of *Fusarium*, which is usually reported in disease studies (67). This pattern may reflect the ecological versatility of *Curvularia*, which is well known for its association with the family Poaceae as host plants. Species of *Curvularia* can function both as foliar pathogens and endophytes. Evidence across different plants, including grasses, highlights that endophytic fungi may enhance host growth and increased resistance to insect pests, pathogens, and herbivores. Such ecological versatility may provide an adaptive advantage, allowing *Curvularia* to colonize and maintain populations within sugarcane stem tissues, supported by its broad geographic distribution (64, 68–70). In addition, reports of *Curvularia* as seed-associated endophytes in sugarcane suggest the possibility of vertical transmission, which may contribute to its stable and recurrent presence in cultivated systems (65, 71). Importantly, the ecological success of these taxa likely reflects their ability to exploit the nutrient-rich parenchyma of sugarcane stems and tolerate the fluctuating moisture and temperature conditions characteristic of field environments (72, 73).

Multilocus phylogenetic analyses provided increased resolution within species complexes known to be taxonomically challenging. For *Curvularia*, the ITS locus alone is insufficient for species delimitation due to intragenomic variation and limited discriminatory power, whereas the combined ITS–GAPDH–TEF1-*α* dataset reliably resolved isolates into *C. plantarum*, *C.* cf. *clavata*, and *C.* cf. *guangxiensis*. These identifications are consistent with recent revisions of the genus, which highlight the utility of TEF1-α and GAPDH as primary barcodes (74). Similarly, *Fusarium* isolates were placed within the *F. fujikuroi* species complex (FFSC), *F. chlamydosporum* species complex (FCSC), and the *F. incarnatum–equiseti* complex (FIESC), in agreement with the well-established association of FFSC members—particularly *F. sacchari*—with sugarcane (31, 67). Our recovery of *Nigrospora zimmermanii* further supports the widespread distribution of *Nigrospora* endophytes in monocot hosts (75).

Mycotoxin profiling revealed a relatively narrow but ecologically meaningful toxigenic landscape. All *F. sacchari* isolates produced BEA, a cyclic hexadepsipeptide commonly associated with FFSC species (76, 77). This metabolite is well-documented to be synthesized by several fungal genera, most notably *Beauveria bassiana*, *Isaria fumosorosea*, and various *Fusarium* species such as *F. proliferatum* and *F. verticillioides* (78–80). The production of BEA in this study is consistent with its known toxigenic potential, as BEA has been previously detected in *Fusarium*-infected sugarcane (23, 81), indicating that this metabolite may represent a consistent biochemical feature of *F. sacchari* populations. Although the concentrations detected in this study were not quantified against calibrated reference curves, the characteristic MRM transitions confirm BEA biosynthesis under laboratory conditions (18). In contrast, CIT production was restricted to *Paecilomyces lecythidis*, a finding of particular significance given that CIT has not been previously associated with this species. Traditionally, members of the genera *Penicillium* (*P. citrinum*, *P. expansum*, and *P. verrucosum*), *Aspergillus* (*A. carneus*, *A. terreus,* and *A. niveus*), and *Monascus* (*M. ruber*, and *M. purpureus*) have been recognized as the principal producers of CIT (82, 83). This represents the first confirmed report of CIT biosynthesis by *P. lecythidis*, expanding the known toxigenic repertoire of the genus and raising questions regarding the ecological role of this metabolite in plant-associated environments. While only two fungal species in this study produced the detected mycotoxins, their absence in the majority of other isolates does not preclude their toxigenic potential under alternative environmental conditions. Beyond the presence of biosynthetic gene clusters, mycotoxin biosynthesis is influenced by multiple factors including substrate composition, water activity, temperature, pH, light, and interspecific microbial interactions (84–86). It is therefore possible that sugarcane tissues provide distinct physicochemical cues that may induce metabolite production under field conditions, but which are not replicated in standard laboratory media.

Compared with other studies, In Southern Iran, representative isolates of four *Fusarium* species complexes from sugarcane were reported to produce fumonisins, BEA, and enniatins (23). In China, *Fusarium* species isolated from leaves affected by Pokkah boeng disease are capable of producing fumonisins, trichothecenes, and zearalenone (ZEA) (87). In Egypt, *F. moniliforme* was identified as a producer of ZEA and diacetoxyscirpenol (DAS) (14). In the present study, the detection of BEA and CIT confirms the toxigenic potential of sugarcane-associated fungi in Thailand. Beyond its insecticidal and antimicrobial activity, BEA is recognized for its cytotoxic, genotoxic, and apoptotic effects (88), while CIT is known for its nephrotoxic and hepatoxic effects (82). Although mycotoxin contamination in sugarcane-derived products was not evaluated in this study, the presence of these mycotoxins suggest potential contamination originating from the sugarcane field, posing health risks to both humans and animals. Consequently, these mycotoxins may persist in the final products or be derived from post-harvest contamination (89). This concern is supported by a study in Brazil, where both aflatoxigenic *Aspergillus* species and aflatoxins (AFs) were detected throughout the production chain, including in sugarcane juice (up to 4.09 μg/kg total AFs) and dried yeast (up to 10.19 μg/kg total AFs) (24). Moreover, a report from India has demonstrated that mycotoxins can persist through industrial processing into jaggery, surviving thermal treatments (90).

To date, there has been no investigation of mycotoxin contamination in sugarcane-derived products in Thailand. In contrast, studies from several countries have confirmed the contamination in sugarcane-derived product such as juice, molasses, jaggery, and ethanol by-products (dry yeast and cream yeast), particularly with AFs (24, 90, 91). Although BEA is a widely prevalent mycotoxin detected in food and feed worldwide (92, 93), such as cereals, rice bran, maize, infant foods, and aquaculture feed (94, 95), and CIT is frequently reported as a contaminant in red yeast rice, cheese, apples, and roasted nuts (82, 96), their occurrence in sugarcane production systems remains largely unexplored as most studies on sugarcane products have primarily focused on AFs. The detection of fungi producing both BEA and CIT in the present study therefore highlights a potential gap in the safety assessment of the Thai sugarcane production chain and underscores the need for comprehensive monitoring of sugarcane-derived product that extends beyond AFs to include these underrecognized metabolites.

Overall, the integrated approach applied in this study provides a foundational understanding of the diversity of fungal isolates recovered from Thai sugarcane stem parenchyma, as well as their mycotoxin production potential. However, these findings should be interpreted within the context of the study’s limitations, including sampling restricted to cultivar Khon Kaen 3 in a single location in Kanchanaburi Province at a single time point, a limited number of isolates subjected to multilocus sequencing, and the targeted screening of 16 selected mycotoxins that may not reflect all secondary metabolites produced. The presence of mycotoxin-producing fungi within stems underscores the need for continuous monitoring of fungal communities—particularly across multiple locations with intensive sugarcane cultivation—to ensure early detection of toxigenic species and to investigate influencing environmental factors. These findings also provide a preliminary scientific basis for future evaluations of mycotoxin occurrence in sugarcane-derived products in Thailand, thereby supporting a more integrated safety framework across the sugarcane production chain. The newly documented toxigenicity of *P. lecythidis* further highlights the importance of evaluating non-traditional genera that may serve as underrecognized contributors to agricultural mycotoxin contamination.

## Data Availability

The original contributions presented in the study are publicly available. This data can be found here: https://www.ncbi.nlm.nih.gov/bioproject/?term=PRJNA1469828. The accession numbers of the fungal isolates included in this study are listed in Table 1 and Supplementary Table 1. The supplementary dataset is also available at: https://doi.org/10.6084/m9.figshare.32387883.
