# Supplementary material for: Integrated polyphasic characterization and mycotoxin production of fungal isolates in sugarcane (Saccharum officinarum) stems from Thailand
Source: Front Nutr. 2026 Jun 10;13:1828952. doi: 10.3389/fnut.2026.1828952 (PMC13292767; doi:10.3389/fnut.2026.1828952)
Supplement: Supplementary file 1 [file Table_1.docx]

**Supplementary Table S1.** Details of *Curvularia* strains included in the phylogenetic analysis.

| **Species** | **Culture accession** | **GenBank accession** | | |
| --- | --- | --- | --- | --- |
|  |  | **ITS** | **GAPDH** | **TEF1-α** |
| *Bipolaris panici-miliacei* | CBS 199.29 LT | KJ909773 | KM042896 | KM093788 |
| *Bipolaris peregianensis* | BRIP 12790 T | JN601034 | JN600977 | JN601022 |
| *Curvularia* sp. | BRIP17086b | MH414904 | MH433648 | MH433666 |
| *Curvularia* sp. 1 | LC12011 | MN215709 | MN264140 | MN264002 |
|  | LC12012 | MN215710 | MN264141 | MN264003 |
| *Curvularia americana* | UTHSC 08 3414 T | HE861833 | HF565488 | – |
| *Curvularia bannonii* | BRIP 16732 IsoT | KJ415542 | KJ415404 | KJ415450 |
|  | DAOM196762 | KP400634 | KP419983 | KP735688 |
| *Curvularia clavata* | BRIP 61680b | KU552205 | KU552167 | KU552159 |
|  | BL3 | KP700959 | KU375468 | – |
|  | BL7 | KP692788 | KU375467 | – |
|  | DAOM148084 | AF071336 | AF081391 | – |
| *Curvularia* cf *clavata* | **SC36 (This study)** | **PX494400** | **-** | **PX829154** |
| *Curvularia elliptiformis* | CGMCC 3.19351 = LC12005 T | MN215656 | MN264091 | MN263950 |
|  | LC12004 | MN215659 | MN264092 | MN263953 |
| *Curvularia eragrostidis* | CBS 189.48 | HG778986 | HG779154 | – |
|  | LZS.52.2 | MH938081 | MH972264 | – |
|  | CD15 | MK886805 | MK886801 | MK886803 |
| *Curvularia graminicola* | BRIP 23186 T | JN192376 | JN600964 | JN601008 |
| *Curvularia guangxiensis* | CGMCC 3.19330 = LC11999 T | MN215667 | MN264100 | MN263961 |
|  | LC11997 | MN215668 | MN264101 | MN263962 |
| *Curvularia* cf *guangxiensis* | **SC48 (This study)** | **PX494399** | **PX762772** | **PX829155** |
| *Curvularia hominis* | CBS 136985 T | HG779011 | HG779106 | – |
| *Curvularia intermedia* | CBS 334.64 | HG778991 | HG779155 | – |
| *Curvularia lonarensis* | CBS 140569 T | KT315408 | KY007019 | – |
| *Curvularia mosaddeghii* | IRAN 3131C T | MG846737 | MH392155 | MH392152 |
| *Curvularia muehlenbeckiae* | CBS 144.63 T | HG779002 | HG779108 | – |
|  | LC11988 | MN215681 | MN264113 | MN263975 |
|  | LC11989 | MN215682 | MN264114 | MN263976 |
|  | LC11990 | MN215683 | MN264115 | MN263977 |
| *Curvularia pandanicola* | MFLUCC 15-0746 T | MH275056 | MH412748 | MH412763 |
| *Curvularia pisi* | CBS 190.48 T | KY905678 | KY905690 | KY905697 |
| *Curvularia plantarum* | CGMCC 3.19342 = LC11986 T | MN215688 | MN264120 | MN263982 |
|  | LC11985 | MN215689 | MN264121 | – |
|  | LC11987 | MN215690 | MN264122 | MN263983 |
|  | **SC53 (This study)** | **PX494398** | **PX762771** | **PX829156** |
| *Curvularia platzii* | BRIP 27703b T | MH414906 | MH433651 | MH433669 |
| *Curvularia polytrata* | CGMCC 3.19338 = LC11991 T | MN215691 | MN264123 | MN263984 |
|  | LC13551 | MN215692 | MN264124 | MN263985 |
| *Curvularia radici foliigena* | CGMCC 3.19328 = LC11957 T | MN215695 | MN264127 | MN263988 |
|  | LC11956 | MN215698 | MN264130 | MN263991 |
| *Curvularia radicicola* | CGMCC 3.19327 = LC11952 T | MN215699 | MN264131 | MN263992 |
|  | LC11953 | MN215700 | MN264132 | MN263993 |
| *Curvularia sacchari officinarum* | CGMCC 3.19331 = LC12006 T | MN215705 | MN264137 | MN263998 |
|  | LC12008 | MN215707 | MN264138 | MN264000 |
| *Curvularia verruculosa* | CBS 150.63 | KP400652 | KP645346 | KP735695 |
|  | MFLUCC 10 690 | JX256437 | JX276448 | JX266602 |
|  | LC11950 | MN215715 | MN264146 | MN264007 |

**Supplementary Table S1.** Details of *Fusarium* strains included in the phylogenetic analysis.

| **Species** | **Culture accession** | **GenBank accession** | | |
| --- | --- | --- | --- | --- |
|  |  | **TEF1-α** | **CAM** | **RPB2** |
| *Fusarium andiyazi* | CBS119856 | MN533989 | MN534174 | MN534286 |
|  | CBS119857T | MN193854 | MN534175 | LT996138 |
| *Fusarium atrivinoum* | CBS445.67T | MN120752 | MN120693 | – |
|  | CBS130394 | MN120753 | MN120694 | MN120734 |
| *Fusarium bubalinum* | CBS161.25=NRRL26857=NRRL26918T | MN170448 | MN170314 | MN170381 |
| *Fusarium caatingaense* | CBS976.97 | MN170449 | MN170315 | MN170382 |
|  | NRRL34003=CBS130317 | GQ505627 | GQ505539 | GQ505805 |
| *Fusarium chlamydosporum* | CBS145.25 = NRRL 26912NT | MN120754 | MN120695 | MN120735 |
|  | CBS677.77 = NRRL 36539 | GQ505422 | GQ505391 | GQ505486 |
|  | CBS615.87 = NRRL 28578 | GQ505405 | GQ505375 | GQ505469 |
|  | **SC41 (This study)** | **PX829161** | **PX829151** | **PX829172** |
| *Fusarium coffeatum* | CBS635.76=BBA62053=NRRL20841T | MN120755 | MN120696 | MN120736 |
|  | NRRL28577 =CBS430.81 | MN120756 | MN120697 | MN120737 |
| *Fusarium concentricum* | CBS450.97T | AF160282 | MW402467 | JF741086 |
|  | CBS453.97 | MN533998 | MN534216 | MN534264 |
| *Fusarium denticulatum* | CBS406.97 | MN533999 | MN534185 | MN534273 |
|  | CBS407.97T | MN534000 | MN534186 | MN534274 |
| *Fusarium fractiflexum* | NRRL28852T | AF160288 | AF158341 | LT575064 |
| *Fusarium fujikuroi* | CBS195.34 | MW402111 | – | – |
|  | CBS221.76T | MN534010 | – | KU604255 |
|  | CBS240.64 | MW402117 | – | – |
| *Fusarium* sp. | NRRL13338 | GQ505402 | GQ505372 | JX171561 |
| *Fusarium* sp. (FIESC22) | NRRL34002 | GQ505626 | GQ505538 | GQ505804 |
| *Fusarium* sp. (FIESC27) | NRRL20722=IMI190455 | GQ505595 | GQ505507 | GQ505773 |
| *Fusarium* sp. (FIESC32) | InaCCF964 | LS479446 | LS479425 | LS479860 |
|  | Indo167 | − | LS479424 | LS479858 |
| *Fusarium guilinense* | NRRL13335=FRCR-2138 | GQ505590 | GQ505502 | GQ505768 |
|  | NRRL32865=FRCR-8480 | GQ505614 | GQ505526 | GQ505792 |
| *Fusarium humicola* | CBS124.73T | MN120757 | MN120698 | MN120738 |
| *Fusarium incarnatum* | CBS132.73=ATCC24387=IMI128222=NRRL25478NT | MN170476 | MN170342 | MN170409 |
|  | CBS132907 | MN170477 | MN170343 | MN170410 |
|  | NRRL13379=FRCR-5198=BBA62200 | GQ505591 | GQ505503 | GQ505769 |
| *Fusarium irregulare* | CBS132190 | MN170480 | MN170346 | MN170413 |
|  | NRRL31160 | GQ505607 | GQ505519 | GQ505785 |
| *Fusarium lactis* | CBS411.97ET | MN193862 | MN534178 | MN534275 |
|  | CBS420.97 | MN534015 | MN534181 | MN534296 |
| *Fusarium luffae* | CBS131097 | MN170482 | MN170348 | MN170415 |
|  | NRRL31167 | GQ505608 | GQ505520 | GQ505786 |
| *Fusarium mangiferae* | CBS119853 | MN534016 | MN534225 | MN534270 |
|  | CBS120994T | MN534017 | MN534224 | MN534271 |
|  | NRRL25226 | AF160281 | AF158334 | HM068353 |
| *Fusarium microconidium* | CBS119843T | MN120759 | MN120700 | – |
| *Fusarium monophialidicum* | NRRL54973 | MN170483 | MN170349 | MN170416 |
| *Fusarium multiceps* | CBS130386=NRRL43639T | GQ505666 | GQ505577 | GQ505844 |
| *Fusarium nelsonii* | CBS119877 | MN120761 | MN120702 | MN120741 |
|  | CBS119876T | MN120760 | MN120701 | MN120740 |
| *Fusarium nygamai* | CBS413.97 | MW402127 | MW402462 | MW402815 |
|  | CBS572.94 | MW402141 | MW402473 | MW402819 |
|  | CBS749.97T | MW402151 | MW402479 | EF470114 |
| *Fusarium pernambucanum* | URM 7559 T | LS398489 | – | LS398519 |
|  | CBS791.70 | MN170491 | MN170357 | MN170424 |
|  | CBS132194 | MN170492 | MN170358 | MN170425 |
|  | **SC52 (This study)** | **PX829162** | **PX829150** | **PX829171** |
| *Fusarium peruvianum* | CBS511.75T | MN120767 | MN120707 | MN120746 |
| *Fusarium phyllophilum* | CBS216.76T | MN193864 | KF466333 | KF466410 |
|  | CBS246.61 | MW402118 | MW402453 | – |
| *Fusarium proliferatum* | CBS480.96ET | MN534059 | MN534217 | MN534272 |
| *Fusarium pseudocircinatum* | CBS449.97T | AF160271 | MN534190 | MN534277 |
|  | CBS455.97 | MN534029 | MN534184 | MN534276 |
| *Fusarium pseudonygamai* | CBS416.97 | MN534030 | MN534194 | MN534283 |
|  | CBS417.97T | AF160263 | AF158316 | MN534285 |
|  | CBS484.94 | MN534031 | MN534195 | MN534284 |
| *Fusarium ramigenum* | CBS418.97T | KF466423 | MN534187 | KF466412 |
|  | CBS526.97 | MN534032 | MN534188 | MN534292 |
| *Fusarium sacchari* | CBS134.73 | MW402041 | – | – |
|  | CBS223.76ET | MW402115 | AF158331 | JX171580 |
|  | CBS186.33 | MW402107 | MW402446 | – |
|  | **SC11 (This study)** | **PX829159** | **PX829148** | **PX829167** |
|  | **SC12 (This study)** | **PX829158** | **PX829149** | **PX829169** |
|  | **SC21 (This study)** | **PX829157** | **PX829147** | **PX829168** |
|  | **SC51 (This study)** | **PX829160** | **PX829146** | **PX829170** |
| *Fusarium spinosum* | NRRL43631 | GQ505427 | – | GQ505491 |
|  | CBS122438T | MN120768 | MN120708 | MN120747 |
| *Fusarium sporodociale* | CBS199.63 | MN120769 | MN120709 | MN120748 |
|  | CBS220.61T | MN120770 | MN120710 | MN120749 |
| *Fusarium sulawesiense* | CBS131.73=ATCC24386=IMI160602=NRRL20425 | MN170500 | MN170366 | MN170433 |
|  | CBS163.57 | MN170501 | MN170367 | MN170434 |
| *Fusarium tanahbumbuense* | CBS145.44=BBA4095 | MN170505 | MN170371 | MN170438 |
|  | CBS131009 | MN170506 | MN170372 | MN170439 |
| *Fusarium thapsinum* | CBS733.97 | MN534043 | MN534191 | JX171600 |
|  | CBS776.96T | MN534044 | – | MN534289 |
| *Fusarium udum* | CBS747.79 | MN193872 | MN534154 | MN534258 |
|  | NRRL25199ET | KY498862 | – | KY498875 |
| *Fusarium verticillioides* | CBS218.76ET | MW402113 | MW402449 | – |
|  | CBS734.97 | MW402146 | AF158315 | EF470122 |
| *Neocosmospora suttonianum* | CBS143214T = NRRL32858 | DQ247163 | MW218092 | EU329630 |
|  | CBS 143224 = NRRL54972 | KC808197 | MW218093 | KC808336 |

**Supplementary Table S1.** Details of *Nigrospora* strains included in the phylogenetic analysis.

| **Species** | **Culture accession** | **GenBank accession** | | |
| --- | --- | --- | --- | --- |
|  |  | **ITS** | **TUB** | **TEF1-α** |
| *Nigrospora aurantiaca* | CGMCC3.18130T | KX986064 | KY019465 | KY019295 |
| *Nigrospora bambusae* | CGMCC3.18327 = LC7114 T | KY385307 | KY385319 | KY385313 |
| *Nigrospora camelliae*-*sinensis* | CGMCC3.18125T | KX985986 | KY019460 | KY019293 |
| *Nigrospora chinensis* | CGMCC 3.18127 T | KX986023 | KY019462 | KY019422 |
|  | LC 4660 | KX986026 | KY019548 | KY019445 |
| *Nigrospora cooperae* | BRIP72440a | OP035048 | OP039540 | OP039539 |
| *Nigrospora covidalis* | CGMCC3.20538 | OK335209 | OK431479 | OK431485 |
| *Nigrospora endophytica* | ARM687 | OM265226 | OP572418 | OP572415 |
| *Nigrospora falsivesicularis* | CGMCC3.19678 = LC12067T | MN215778 | MN329942 | MN264017 |
| *Nigrospora globospora* | CGMCC3.20539 | OK335211 | OK431481 | OK431487 |
| *Nigrospora gorlenkoana* | CBS480.73T | KX986048 | KY019456 | KY019420 |
| *Nigrospora guilinensis* | CGMCC3.18124 = LC3481 | KX985983 | KY019459 | KY019292 |
| *Nigrospora hainanensis* | CGMCC3.18129 = LC7030 | KX986091 | KY019464 | KY019415 |
|  | LC13514 | MN215780 | MN329944 | MN264019 |
|  | LC13515 | MN215781 | MN329945 | MN264020 |
| *Nigrospora lacticolonia* | CGMCC3.18123 = LC3324 | KX985978 | KY019458 | KY019291 |
| *Nigrospora marylouisemclawsiae* | BRIP74865b | PP125567 | PP209362 | PP209361 |
| *Nigrospora mercuriadeae* | BRIP75764a | PP707904 | PP712794 | PP712793 |
| *Nigrospora musae* | CBS319.34T | KX986076 | KY019455 | KY019419 |
|  | LC6385 | KX986042 | KY019567 | KY019371 |
| *Nigrospora oryzae* | LC6761 | KX986056 | KY019574 | KY019376 |
|  | LC6759 | KX986054 | KY019572 | KY019374 |
| *Nigrospora osmanthi* | CGMCC3.18126T | KX986010 | KY019461 | KY019421 |
| *Nigrospora philosopgiae*-*doctoris* | CGMCC3.20540 | OK335213 | OK431483 | OK431489 |
| *Nigrospora pyriformis* | CGMCC3.18122T | KX985940 | KY019457 | KY019290 |
| *Nigrospora rubi* | CGMCC3.18326 = LC2698 | KX985948 | KY019475 | KY019302 |
| *Nigrospora saccharicola* | CGMCC3.19362 = LC12056T | MN215788 | MN329951 | MN264027 |
| *Nigrospora sacchari*-*officinarum* | CGMCC3.19335 = LC12076T | MN215791 | MN329954 | MN264030 |
|  | LC13531 | MN215792 | MN329955 | MN264031 |
| *Nigrospora singularis* | CGMCC3.19334 = LC12069T | MN215793 | MN329956 | MN264032 |
| *Nigrospora sphaerica* | LC2840 | KX985965 | KY019492 | KY019318 |
|  | LC13527 | MN215809 | MN329972 | MN264048 |
|  | LC7312 | KX985935 | KY019618 | KY019414 |
|  | LC2705 | KX985952 | KY019479 | KY019305 |
| *Nigrospora stoneae* | BRIP75022a | OR608744 | OR604067 | OR604065 |
| *Nigrospora vesicularifera* | CGMCC3.19333 = LC12052T | MN215812 | MN329975 | MN264051 |
| *Nigrospora vesicularis* | CGMCC3.18128 = LC7010 | KX986088 | KY019463 | KY019294 |
| *Nigrospora zimmermanii* | CBS290.62T | KY385309 | KY385317 | KY385311 |
|  | LC12048 | MN215818 | MN329981 | MN264057 |
|  | LC13536 | MN215819 | MN329982 | MN264058 |
|  | LC13535 | MN215820 | MN329983 | MN264059 |
|  | LC12050 | MN215821 | MN329984 | MN264060 |
|  | LC13537 | MN215822 | MN329985 | MN264061 |
|  | LC12051 | MN215823 | MN329986 | MN264062 |
|  | LC13534 | MN215824 | MN329987 | MN264063 |
|  | **SC23 (This study)** | **PX494401** | **PX829177** | **PX829163** |
|  | **SC26 (This study)** | **PX494402** | **PX829178** | **PX829164** |
