# Supplementary material for: Integrated polyphasic characterization and mycotoxin production of fungal isolates in sugarcane (Saccharum officinarum) stems from Thailand
Source: Front Nutr. 2026 Jun 10;13:1828952. doi: 10.3389/fnut.2026.1828952 (PMC13292767; doi:10.3389/fnut.2026.1828952)
Supplement: Supplementary file 2 [file Table_2.docx]

**Supplementary Table S2.** MS/MS parameters for the determination of 16 type mycotoxins.

| Analyte | Precursor ion (m/z) | Product ions (m/z) | Collision energy (eV) | Fragmentor (V) | Retention time (min) | Polarity |
| --- | --- | --- | --- | --- | --- | --- |
| OTA | 404.00 | 192.9  102.1^a^ | 48  80 | 130  130 | 6.79 | Positive |
| ZEA | 319.16 | 283.0  187.0^a^ | 5  17 | 80  80 | 7.24 | Positive |
| BEA | 801.40 | 784.3  244.1^a^ | 13  35 | 160  160 | 8.43 | Positive |
| FB1 | 722.40 | 352.5  334.4^a^ | 40  45 | 160  160 | 5.59 | Positive |
| FB2 | 706.30 | 336.2  318.3^a^ | 35  40 | 200  200 | 5.99 | Positive |
| T2 | 489.40 | 387.3  245.2^a^ | 20  26 | 170  170 | 7.00 | Positive |
| ALT | 259.10 | 187.9  160.1^a^ | 25  33 | 240  240 | 6.08 | Positive |
| CIT | 251.10 | 233.1  205.1^a^ | 3  20 | 120  120 | 6.75 | Positive |
| ENN A | 699.3 | 682.3  209.7 | 14  30 | 160  160 | 8.095 | Positive |
| ENN A1 | 668.5 | 201.1  196.1 | 25  25 | 160  160 | 7.963 | Positive |
| ENN B | 640.4 | 214.1  196.1 | 25  25 | 160  160 | 7.709 | Positive |
| ENN B1 | 654.4 | 637.3  581.2 | 13  25 | 160  160 | 7.797 | Positive |
| STER | 325.1 | 309.9  281 | 25  35 | 160  160 | 8.628 | Positive |
| PAT | 153.0 | 109.0  81.0 | 1  4 | 160  160 | 6.35 | Positive |
| DON | 355.10 | 265.1  59.1^a^ | 4  10 | 90  90 | 3.71 | Negative |
| NIV | 371.10 | 281.0  59.1^a^ | 4  10 | 80  80 | 2.26 | Negative |
