# Supplementary material for: Integrated polyphasic characterization and mycotoxin production of fungal isolates in sugarcane (Saccharum officinarum) stems from Thailand
Source: Front Nutr. 2026 Jun 10;13:1828952. doi: 10.3389/fnut.2026.1828952 (PMC13292767; doi:10.3389/fnut.2026.1828952)
Supplement: Supplementary file 3 [file Table_3.docx]

**Supplementary Table S3.** Performance characteristics of the proposed method

| Analyte | **Culture medium** | | |
| --- | --- | --- | --- |
|  | LOD  (µg/kg) | LOQ  (µg/kg) | R^2^ |
| OTA | 0.19 | 0.62 | 0.9995 |
| ZEA | 7.73 | 25.5 | 0.9980 |
| BEA | 0.15 | 0.51 | 0.9996 |
| FB1 | 0.38 | 1.25 | 0.9975 |
| FB2 | 0.53 | 1.77 | 0.9985 |
| T2 | 0.98 | 3.26 | 0.9985 |
| DON | 8.47 | 28.2 | 0.9965 |
| NIV | 25.6 | 85.3 | 0.9970 |
| ALT | 0.56 | 1.88 | 0.9991 |
| CIT | 0.67 | 2.23 | 0.9992 |
| ENN A | 0.02 | 0.08 | 0.9955 |
| ENN A1 | 0.03 | 0.10 | 0.9984 |
| ENN B | 0.04 | 0.12 | 0.9975 |
| ENN B1 | 0.04 | 0.12 | 0.9968 |
| STER | 0.08 | 0.25 | 0.9895 |
| PAT | 0.06 | 0.20 | 0.9993 |
